# Antibacterial property of Ag nanoparticle-impregnated N-doped titania films under visible light

**DOI:** 10.1038/srep11978

**Published:** 2015-07-09

**Authors:** Ming-Show Wong, Chun-Wei Chen, Chia-Chun Hsieh, Shih-Che Hung, Der-Shan Sun, Hsin-Hou Chang

**Affiliations:** 1 Department of Materials Science and Engineering, National Dong Hwa University, Hualien, Taiwan; 2 Nanotechnology Research Center, National Dong Hwa University, Hualien, Taiwan; 3 Department of Molecular Biology and Human Genetics, Tzu Chi University, Hualien, Taiwan; 4 Research Center of Nanobiomedical Science, Tzu Chi University, Hualien, Taiwan

## Abstract

Photocatalysts produce free radicals upon receiving light energy; thus, they possess antibacterial properties. Silver (Ag) is an antibacterial material that disrupts bacterial physiology. Our previous study reported that the high antibacterial property of silver nanoparticles on the surfaces of visible light-responsive nitrogen-doped TiO_2_ photocatalysts [TiO_2_(N)] could be further enhanced by visible light illumination. However, the major limitation of this Ag-TiO_2_ composite material is its durability; the antibacterial property decreased markedly after repeated use. To overcome this limitation, we developed TiO_2_(N)/Ag/TiO_2_(N) sandwich films in which the silver is embedded between two TiO_2_(N) layers. Various characteristics, including silver and nitrogen amounts, were examined in the composite materials. Various analyses, including electron microscopy, energy dispersive spectroscopy, X-ray diffraction, and ultraviolet–visible absorption spectrum and methylene blue degradation rate analyses, were performed. The antibacterial properties of the composite materials were investigated. Here we revealed that the antibacterial durability of these thin films is substantially improved in both the dark and visible light, by which bacteria, such as *Escherichia coli, Streptococcus pyogenes, Staphylococcus aureus,* and *Acinetobacter baumannii*, could be efficiently eliminated. This study demonstrated a feasible approach to improve the visible-light responsiveness and durability of antibacterial materials that contain silver nanoparticles impregnated in TiO_2_(N) films.

Disinfectants are crucial for reducing pathogenic microorganisms for personal hygiene and in water treatment, food production, and health care facilities^1^. Titanium dioxide (TiO_2_) substrates are the most frequently used photocatalyst for antibacterial purposes^2^. After exposure to ultraviolet (UV) light, the photon energy excites electrons from the valence band to the conduction band, leaving positive holes in the valence band. The electrons and holes may recombine, releasing energy in the form of light or heat, causing inefficient photocatalysis. However, the excited electrons and holes may be trapped on or near the TiO_2_ surface and subsequently react with atmospheric water and oxygen, yielding reactive oxygen species (ROS) such as hydroxyl radicals (•OH) and superoxide anions (O_2_^−^)^3^. These ROS aid in eliminating pathogenic microorganisms; however, UV irradiation is hazardous to humans. Therefore, UV-responsive photocatalysts, such as TiO_2_, are unsuitable for use in living environments.

To avoid this problem, impurity may be doped in TiO_2_ films with carbon, sulfur, nitrogen (N), or silver (Ag) results in an excitation wavelength shift from UV to the visible light region^4^,^5^,^6^,^7^. In addition, impurity doping or metal-particle-impregnated TiO_2_ may reduce the recombination rates of pairs of electrons and holes. Furthermore, impregnated material such as silver possesses antibacterial properties. Therefore, visible-light-responsive antibacterial photocatalysts, which ensure a high quantum efficiency under sunlight and can be used safely in indoor settings to prevent users from being exposed to biohazardous UV light, have been developed^2^,^5^,^6^,^8^,^9^,^10^,^11^,^12^,^13^.

Our previous study indicated that nitrogen-doped TiO_2_ [TiO_2_(N)] thin film coated with silver nanoparticles exhibited higher antibacterial activity under the illumination of visible light^6^. However, the major limitation of this film is its durability. Because the silver nanoparticles are directly exposed to the environment and easily detached from the surfaces of the thin films, we observed that the antibacterial activity was substantially reduced after repeated usages. This demonstrates the need for a multilayer or sandwich thin film in which the silver is embedded between the two TiO_2_(N) layers of thin films.

The concept of multilayered TiO_2_/Ag films has been described in research for decades, with various purposes and rationales provided. For example, transparent heat‐mirror films of TiO_2_/Ag/TiO_2_ were proposed for solar energy collection and radiation insulation^14^ and transparent conductive coatings of TiO_2_/Ag/TiO_2_^15^ were also reported. The multilayered TiO_2_/Ag films described in the literature enhance photocatalysis under UV irradiation^16^. However, visible-light-responsive Ag-TiO_2_ sandwich films with higher durability and effective antibacterial properties are yet to be developed. Previous studies have described the development of visible-light-responsive antibacterial photocatalysts, which ensure high quantum efficiency under sunlight and can be used safely in indoor settings, preventing users from UV exposure, as a major advancement in photocatalytic material research^2^,^5^,^6^,^8^,^9^,^10^,^11^,^12^,^13^. In addition, most antibacterial studies have employed normal bacterial flora, such as *E. coli*, which may not reflect the real targets of human pathogens. Notably, antibacterial materials may apply to particular type (e.g. Gram-negative bacteria^17^) but not the other types of bacteria. Therefore, in this study, we developed various novel Ag-TiO_2_ sandwich films with a high visible-light-responsiveness and then investigated their antibacterial properties. In addition to *E. coli*, pathogenic bacteria, such as *S. aureus*, *S. pyogenes* (Gram-positive), and *A. baumannii* (Gram-negative), were first used to analyze the bactericidal effects of these Ag-TiO_2_ sandwich films.

## Results

### Effects of silver level on photocatalytic and antibacterial properties of TiO_2_(N)/Ag/TiO_2_(N) sandwich films

**Figure 1:**
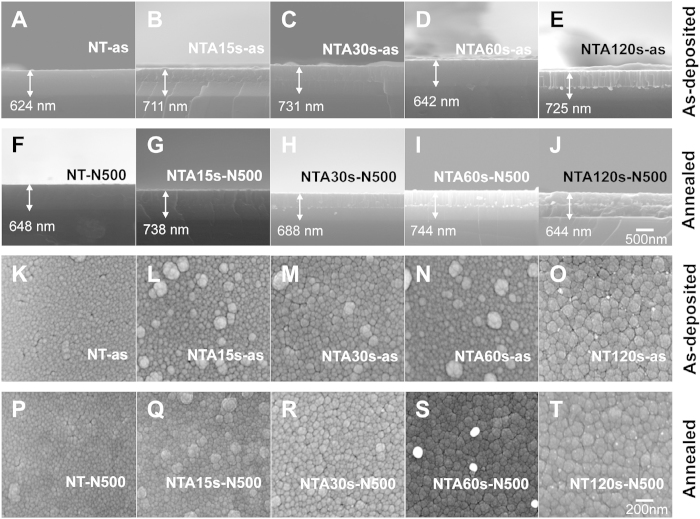
Field emission scanning electron microscopy of TiO_2_(N)/Ag/TiO_2_(N) films. Images of lateral view (**A**–**J**) and vertical view (**K–T**) of various amount of Ag sandwiched in N-doped TiO_2_ thin films before (**A–E**, **K–O**) and after (**F–J**, **P–T**) annealing were showed. The thin films formed in TiO_2_(N)/Ag/TiO_2_(N) sandwich structure as indicated in the Supplementary Fig. S5.

**Figure 2:**
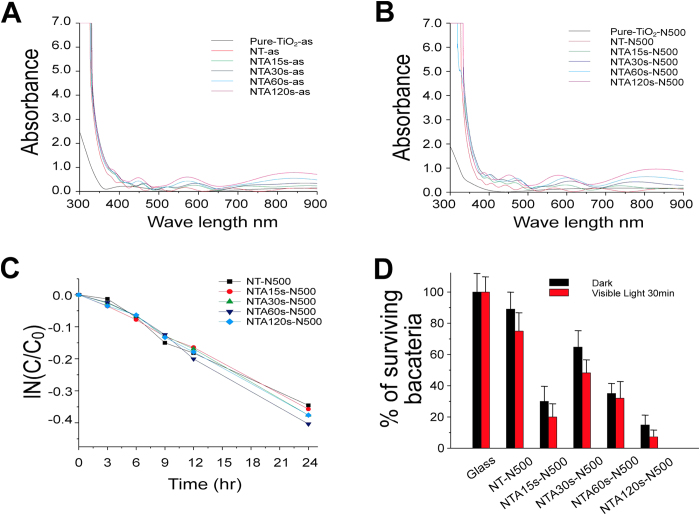
Influence of silver amount in TiO_2_(N)/Ag/TiO_2_(N) films. UV-visible absorption spectroscopy of samples before (**A**) and after (**B**) annealing, methylene blue degradation (**C**) and bacterial survival (CFU) (**D**) were shown. Control groups on the glass were normalized to 100%. There are no statistical significances between visible-light and dark groups. *n* = 3.

To regulate the amount of Ag deposition in the middle layer of sandwich films, various sputtering periods, namely 0, 15, 30, 60, and 120 s, were examined. X-ray diffraction patterns of the sandwich films revealed that the anatase phase of titania was formed after annealing (Supplementary Fig. S1). Energy-dispersive X-ray spectroscopy analysis indicated that the amount of Ag deposition increased with time (Supplementary Fig. S2). Furthermore, a field-emission scanning electron microscopy analysis revealed that the thickness of the sandwiched thin films was nearly 700 ± 50 nm, in which the thickness for the silver layers are 1.9, 3.8, 7.5 and 15 nm for the specimens prepared under various sputtering time periods of 15, 30, 60, 120 s, respectively (Fig. 1A–J). In addition, various amounts of Ag nanoparticles were observed on the TiO_2_(N) surfaces of some films before and after annealing (Fig. 1K–T, white spots). An ultraviolet–visible (UV–Vis) absorption spectroscopy analysis revealed no significant difference after annealing; whereas the Ag-containing samples showed a redshift, indicating an increased visible light absorption (Fig. 2A,B). The visible-light-responsive photocatalytic property was determined using a methylene blue (MB) degradation analysis as described^18^. Data revealed that Ag doping did not markedly increase the visible-light-responsive photocatalysis (Fig. 2C). Despite this, the antibacterial property was determined. Because of the lack of the visible-light-induced photocatalytic activity, no significant difference was observed between the groups with or without visible light illumination (Fig. 2D). Because these thin films contained Ag, the antibacterial property was detectable even in the dark conditions. The NTA120s sample, which contained the highest Ag levels (Supplementary Fig. S1) exhibited the most antibacterial activity compared with the other tested samples (Fig. 2D).

### Effect of nitrogen level on photocatalytic and antibacterial properties of TiO_2_(N)/Ag/TiO_2_(N) sandwich films

**Figure 3:**
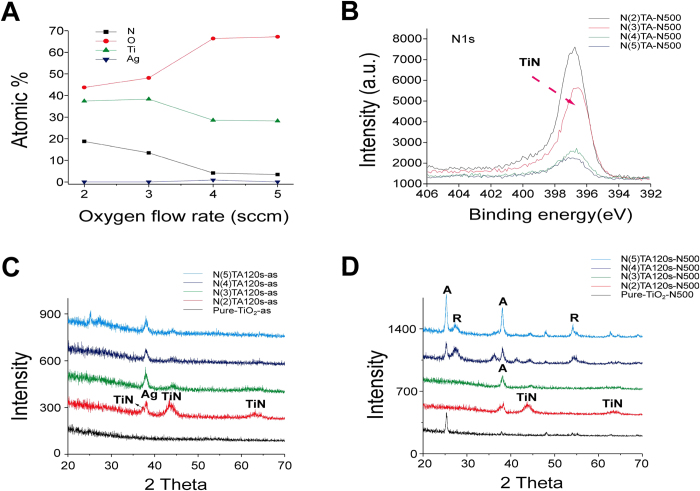
Influence of nitrogen content in TiO_2_(N)/Ag/TiO_2_(N) films. The X-ray photoelectron spectroscopy (XPS) of the surface composition of the films (**A**), XPS analysis for the 1 s atomic orbital of N (**B**) and the XRD analyses before (**C**) and after (**D**) annealing were shown. The number “*x*” labeled in the brackets of “N(*x*)TA” indicated the O_2_ flow rates during the sputtering processes (standard cubic centimeters per minute; sccm).

**Figure 4:**
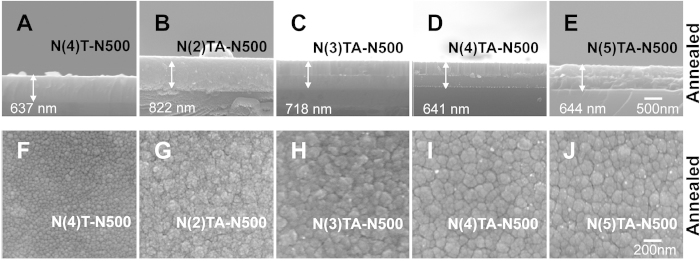
Field emission scanning electron microscopy of TiO_2_(N)/Ag/TiO_2_(N) films of various nitrogen content. Images of lateral view (**A–E**) and vertical view (**F–J**) of TiO_2_(N)/Ag/TiO_2_(N) films after annealing were showed.

**Figure 5:**
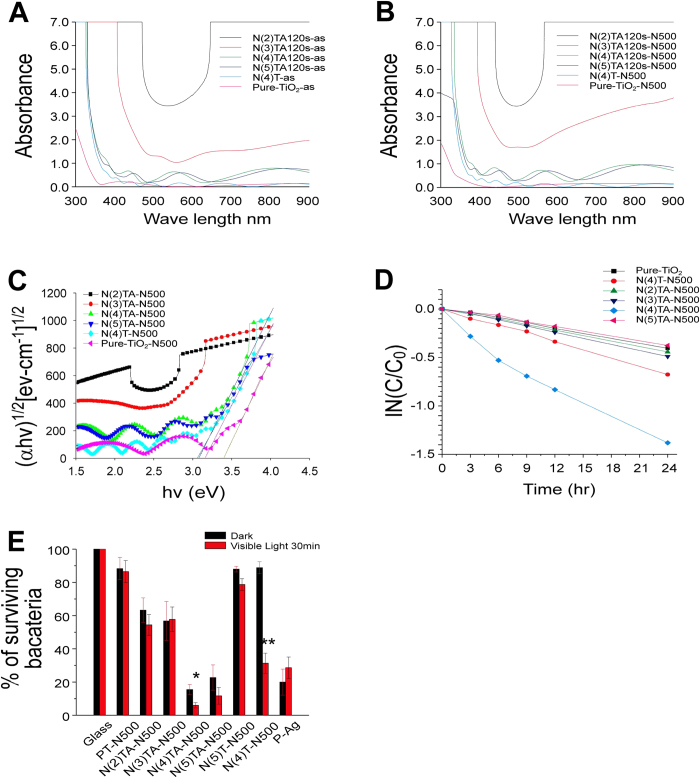
Influence of nitrogen content in TiO_2_(N)/Ag/TiO_2_(N) on the film property. Analyses of UV-visible absorption spectroscopy before (**A**) and after (**B**) annealing, the band gaps (**C**) the methylene blue degradation (**D**) and the bacterial survival (CFU) (**E**) were shown. Control groups on the glass were normalized to 100%. *P < 0.05, **P < 0.01, compared to respectively dark groups. *n* = 3.

To increase the visible-light responsiveness, N doping is critical^6^. The nitrogen content in these films was minimal when oxygen flow was high [5 sccm (standard cubic centimeters per minute)] during film deposition. We hypothesized that this was caused by an excess supply of oxygen, rendering N unable to be efficiently integrated into the lattice structure of TiO_2_. Thus, various low-O_2_-supplying conditions, including the O_2_ flow rate of 2, 3, 4, and 5 sccm, were tested (Supplementary Table 1). Consistent with our hypothesis, an X-ray photoelectron spectroscopy analysis revealed that reduced O_2_ supply improved N levels in composite thin films (Fig. 3A). However, the Ti-to-O ratio was also changed, with the N(4)TA and N(5)TA samples exhibiting a relatively 1:2 ratio, whereas the N(2)TA and N(3)TA samples exhibited a ratio of approximately 1:1 (Fig. 3A; the amount of oxygen supplied was indicated in brackets, e.g., N(4)TA was prepared with 4 sccm O_2_). These results indicated that 2 and 3 sccm O_2_ supply were insufficient to oxidize Ti and produce TiO_2_; instead, TiN or TiON was formed. Consistently, X-ray photoelectron spectroscopy analysis for the 1 s atomic orbital of N indicated a significant increase in the formation of TiN (Fig. 3B). X-ray diffraction analysis further revealed the production of TiN in N(2)TA and N(3)TA samples before and after annealing (Fig. 3C,D). Because TiO_2_ was not efficiently produced in the N(2)TA and N(3)TA samples, the rutile and anatase TiO_2_ signals were observed only in the N(4)TA and N(5)TA thin films after annealing (Fig. 3D). The field-emission scanning electron microscopy analysis indicated that the N(2)TA and N(3)TA samples formed thicker films, with less surface-exposed Ag nanoparticles compared with the N(4)TA and N(5)TA samples (Fig. 4A–E). A UV–Vis absorption spectroscopy analysis indicated that reduced O_2_ supply caused remarkable redshifts in the samples (Fig. 5A,B). The band gaps were calculated using the UV–Vis spectroscopy and Tauc plots^19^. However, the band gaps for the N(2)TA and N(3)TA were not derived, because the two samples produced under 2 and 3 sccm O_2_ supply were insufficient to form TiO_2_; instead, TiN or TiON was formed with high amount of nitrogen of 18.8% and 13.5% and thus, with high absorbance (Fig. 3A,B). These results indicated that the increased N content reduced the band gap of the thin films (Fig. 5C, Supplementary Table S1). Analyses of MB and Hoechst dye degradation rates further revealed that the N(4)TA sample exhibited a superior visible-light-induced photocatalytic ability than did the other samples (Fig. 5D; Supplementary Fig. S3), suggesting that the balance of the N-doping amount and TiO_2_ production was critical. In accordance with the aforementioned analyses, antibacterial experiments demonstrated that the N(4)TA sample exhibited the highest antibacterial activity among the thin film samples under visible light illumination (Fig. 5E, N(4)TA-N500). In addition, visible light illuminating the N(4)TA sample exerted higher bactericidal activity than did the control groups with the N(4)TA sample in the dark (Fig. 5E, N(4)TA-N500; *P < 0.05).

### Durability of visible-light-responsive antibacterial property

**Figure 6:**
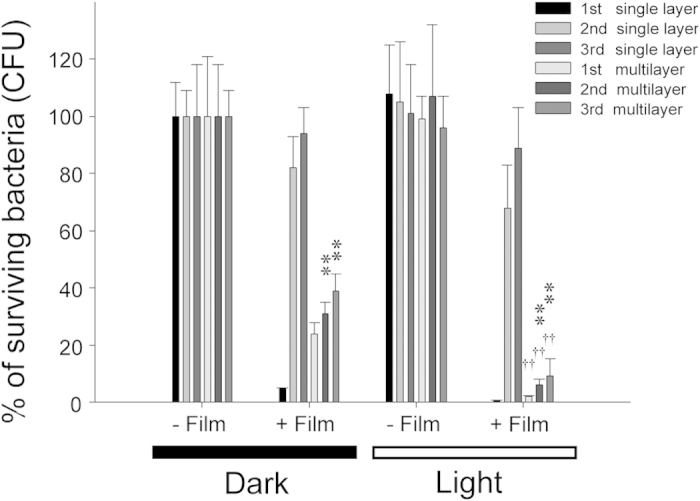
Bactericidal activity analysis after repeated use. Bactericidal activities of the single layer or sandwich (multilayer) thin films with (light) or without (dark) visible-light illumination after used for 1 (1 st), 2 (2nd) and 3 (3rd) times were shown. Respective single layer-dark groups were normalized to 100%. ^††^*P* < 0.01 compared to respective dark groups; **P < 0.01 compared to respective single layer groups.

**Figure 7:**
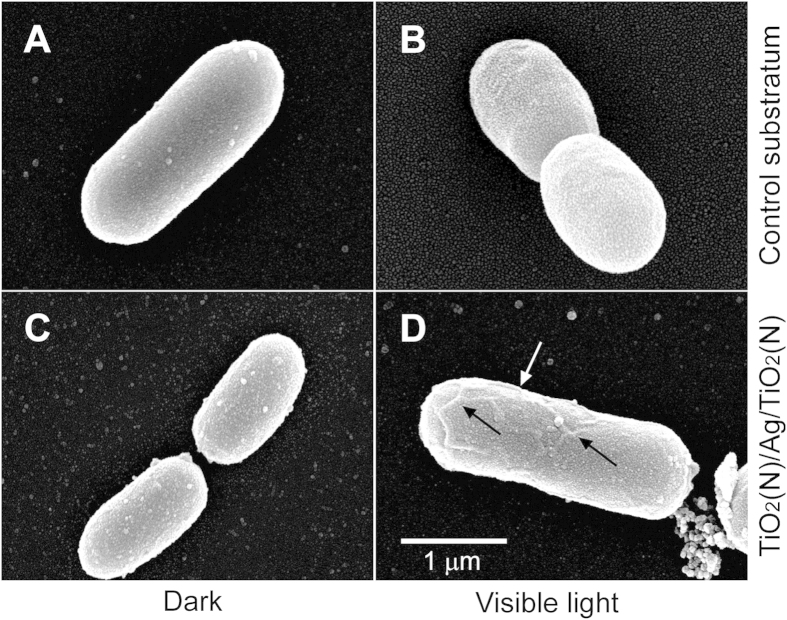
Scanning electron microscopy. The morphology of *E. coli* cells before (**A**,**C**; dark) and after (**B,D**) subjected to visible-light driven photoinactivation on control substratum (**A,B**) or on TiO_2_(N)/Ag/TiO_2_(N) films (**C,D**).

The visible-light-responsive antibacterial property of these novel sandwich films after repeated usages was compared with that of our previously developed single-layer thin films containing Ag nanoparticles^6^. Although the first use of the single-layer thin films could achieve an approximately 5-log reduction in bacteria^6^, the data indicated that these thin films could not efficiently eliminate the bacteria after frequently use (Fig. 6, second- and third-cycle use of single-layer groups). By contrast, favorable performance was observed after these multilayer films were repeated used, which eliminated *E. coli* efficiently after the second and third use (Fig. 6, second- and third-cycle use of multilayer groups). Scanning electron microscopy was employed to investigate the *E. coli* cell damage caused by the sandwich films. We observed that if the bacterial cells were not treated with Ag present in the N-doped TiO_2_ sandwich films with or without visible light illumination, they displayed relatively smooth surfaces (Fig. 7A,B). Because of the antibacterial property of Ag, bacterial cells displayed rough surfaces after being treated with a sandwich film in the dark (Fig. 7C). More vigorous changes, which were showed as unique cracks, such as structures on their surfaces, were observed after the bacterial cells were illuminated with visible light on a sandwich film (Fig. 7D, arrows).

### Pathogen analyses

**Figure 8:**
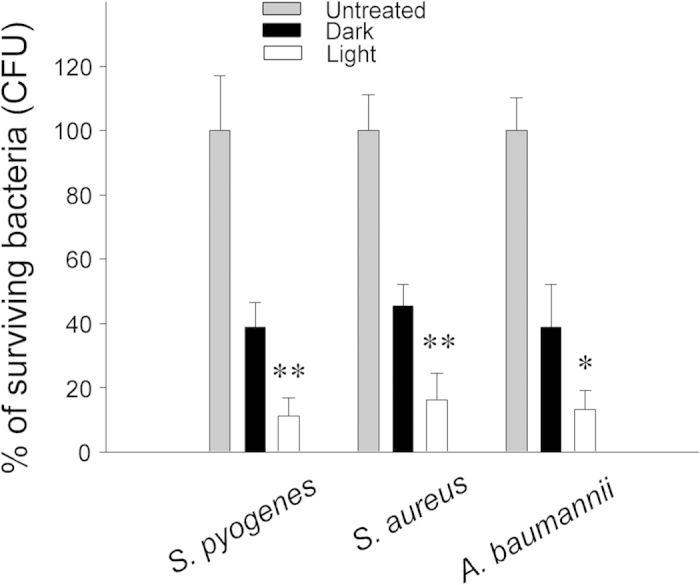
Pathogen analysis of the TiO_2_(N)/Ag/TiO_2_(N) films. For each pathogen, the percentage of surviving bacteria on the control substratum (untreated) was normalized to 100%. **P < 0.01 (compared to respective dark group).

To investigate the performance of TiO_2_(N)/Ag/TiO_2_(N) sandwich films in eradicating pathogenic bacteria and human pathogens, including *S. pyogenes*, *S. aureus*, and *A. baumannii*, the films were subjected to visible-light-induced catalysis. Among these, *S. aureus* and *A. baumannii* are pathogenic bacteria with a high antibiotic resistance rate, causing increased incidence of nosocomial infections^20^. We demonstrated that all the tested pathogens were efficiently eliminated after exposure to the sandwich films illuminated with visible light (Fig. 8, dark vs. light groups). The effectiveness showed a nearly 1-log reduction of the bacterial population. In addition, the TiO_2_(N)/Ag/TiO_2_(N) sandwich films appeared equally potent in eliminating the bacteria when applied to both the Gram-positive (*S. aureus* and *S. pyogenes*) and Gram-negative (*E. coli* and *A. baumannii*) bacteria (Fig. 8).

## Discussion

Silver has been used to prevent infections for thousands of years^21^, with Hippocrates describing the antimicrobial properties of the metal in 400 BC. Antibacterial silver products have been widely used for the handling and cleaning of burn, trauma, catheter, and dental amalgam^22^,^23^. Previous studies have demonstrated that both Ag^+^ ions and Ag nanoparticles possess antibacterial properties^22^,^23^,^24^. Silver disrupts various bacterial physiologies, including disulfide bond formation, metabolism, and iron homeostasis; these changes increase production of ROS and membrane permeability and disrupt membrane respiratory electron transport chains and DNA replication components^23^,^25^. Thus, silver is widely used as tableware and a hygiene product. In these cases, the durability of the antibacterial property is not a problem because the entire metal product is able to release a sufficient amount of Ag ions and nanoparticles with time. However, this is not the case when only a nanoscaled layer of silver is used.

Previous studies have indicated that Ag particles or their coating on TiO_2_ surfaces can enhance the light-driven photoinactivation of bacteria^2^,^6^,^26^,^27^,^28^,^29^,^30^. However, the antibacterial activity of such thin films after repeated use has not been investigated thoroughly. In addition, the antibacterial property of Ag-TiO_2_ composite thin films remains to be elucidated. For example, our previous study reported that TiO_2_ containing nanoscale Ag wires exhibited strong visible-light enhanced antibacterial properties^6^. The durability of these thin films is always a problem; because their antibacterial properties rapidly decline after repeated use (Fig. 6, second- and third-cycle use of single-layer groups). Therefore, we described the influence of the various amounts of silver and nitrogen on the antibacterial property of the sandwich films. Analysis results indicated that silver depositions improved the antibacterial activity of the thin films (Fig. 2). In addition, the amount of nitrogen supplied is crucial for the formation of high-performance antibacterial thin films under visible light illumination (Fig. 5). Thus, we developed the TiO_2_(N)/Ag/TiO_2_(N) sandwich films, which exhibited sustainable antibacterial properties after repeated use.

Nanoscaled thin films have been used to achieve a controlled release of embedded materials^31^,^32^,^33^. In the original experimental design, the silver layer was embedded in the middle of TiO_2_(N)/Ag/TiO_2_(N) sandwich films. Notably, in addition to the embedded Ag layer, we observed certain scattered Ag nanoparticles on the surface of the as-deposited sandwich films and a higher density of Ag nanoparticles on annealed films. Ag nanoparticles appeared to emerge on the film surface by atomic diffusion through the grain boundaries of the upper TiO_2_(N) layer. In addition, this could happen when silver atoms gain sufficient energy from the bombardment of atoms and ions during sputtering growth or from thermal annealing. In either case, the Ag nanoparticles are impregnated in the TiO_2_(N) films and not merely placed or attached on the film surface, which could be the main reason for the high durability of the photocatalytic TiO_2_(N)/Ag/TiO_2_(N) sandwich films.

The silver-induced bactericidal effect is a complex response, involving the disruption of bacterial physiologies and abnormal elicitation of ROS in bacteria^23^,^25^,^34^. Combined treatment with exogenous ROS and silver revealed a synergistic antibacterial effect^35^. ROS production is the main antibacterial mechanism of photocatalysts^2^,^36^,^37^. Thus, it is reasonable that the silver synergizes with the photocatalytic components of the sandwich films to efficiently eradicate pathogenic bacteria under the visible light (Fig. 8). However, nanoscale Ag particles and TiO_2_ appear to exert a synergistic impact on the environment^38^,^39^. The increased production and use of silver nanoparticles in products leads to the inevitable increase in the release of these particles into the environment through the lives of these products, from the raw material stage to disposal^40^. Likewise, the increased disposal of TiO_2_ nanoparticles exerts a considerable impact on the ecosystem^41^. Although the mechanism remains to be further elucidated, the coexistence of Ag and TiO_2_ nanoparticles has been shown to exert increased environmental impact under sunlight^38^,^39^. Furthermore, exposure to Ag nanoparticles was shown to influence the immune system^42^. However, previously reported Ag–TiO_2_ thin films have typically had a surface-exposed Ag layer^6^,^43^,^44^,^45^,^46^. Thus, the increase reusability of the nanoscaled Ag-TiO_2_ composite material with a TiO_2_-covered layer will reduce the environmental impact of products and will benefit human health.

In conclusion, we successfully demonstrated the antibacterial properties of TiO_2_(N)/Ag/TiO_2_(N) sandwich films, which could be optimized through Ag and N depositions on titania substrates. The silver and TiO_2_(N) composite materials exhibited synergistic antibacterial activity under visible light illumination. These findings suggest that the concepts used in this study and multilayer TiO_2_(N)/Ag/TiO_2_(N) composite materials have potential applications in the developing alternative disinfectants.

## Methods

### Various thin films, including single layers of Ag, TiO_2_, and TiO_2_(N), and TiO_2_(N)/Ag/TiO_2_(N) sandwich layers were prepared in a reactive magnetron sputtering system

As Supplementary Fig. S4 illustrates, five sputtering targets exist in the deposition system, but only two titanium (Ti) targets and one silver (Ag) target were used, and the base pressure was below 1.3 × 10^−5^ Pa. To prepare pure TiO_2_ or TiO_2_(N) and Ag, two Ti targets and one Ag target were used, respectively. Before sputtering, we used argon plasma to etch the surface of the substrate for 10 min to remove residual particles on the surface. The substrate holder was rotated at a speed of 5 rpm without applying substrate bias during deposition. Silicon wafer (100) and glass substrates were used. The substrate was nearly at room temperature without external heating. The target powers were set at 250 W each and 20 W in the DC mode for the two Ti targets and one Ag target, respectively. For pure TiO_2_ deposition, the gas contained argon and oxygen with the fixed flow rates of Ar at 20 sccm and O_2_ at 7 sccm, and the total pressure was approximately 4.7 × 10^−1^ Pa (3.5 × 10^−3^ Torr). For the TiO_2_(N) films, the gas contained Ar, O_2_, and N_2_ at the flow rates of 20, 5–2, and 8 sccm, respectively, whereas for the Ag film, the gas used contained only Ar with a flow rate of 20 sccm without adding other gases.

In addition, for forming a monolithic layer of Ag, TiO_2_, and TiO_2_(N), two series of sandwiched TiO_2_(N)Ag/TiO_2_(N) films were prepared. Furthermore, one series was prepared with various amounts of Ag in the sandwiched films, whereas the other series was prepared with a fixed amount of Ag but with various nitrogen contents in the sandwiched films. In the first series, the amount of Ag was regulated by changing the deposition time from 30 s to 60, 90, and 120 s, and the corresponding sample IDs were denoted as NTAxxxs, where xxx was the Ag deposition time. In the second series, the amount of Ag was fixed with the deposition time of 120 s, and the nitrogen contents in the TiO_2_(N) films were regulated by reducing the oxygen flow rates from 5 sccm to 4, 3, and then 2 sccm, and the corresponding sample IDs were denoted as N (x)TA, where x was the oxygen flow rate. The as-deposited films were further annealed at 500 °C for 1 h in the nitrogen atmosphere by using a conventional vacuum furnace.

The deposition rate of TiO_2_(N) films varied with the oxygen flow rate and was approximately 350 nm/h with the oxygen flow rate of 4 sccm. The deposition rate of silver was approximately 7.5 nm/min. The deposition times of the typical sandwich TiO_2_(N)/Ag/TiO_2_(N) film used in this study were 1 h and 2 min, and 1 h. Thus, an average thickness of the Ag impregnated TiO_2_(N) films was 700 ± 50 nm. The thickness for the silver layer are 1.9, 3.8, 7.5 and 15 nm for the specimens prepared under various sputtering time periods of 15, 30, 60, 120 s, respectively.

### Characterization analysis

The structure and crystallinity of films were analyzed using X-ray diffraction measurements recorded using the Rigaku X-ray diffractometer D/MAX-2500 V with a Cu Kα radiation (40 kV, 100 mA) source. The surface morphology and the cross-sectional view of the films were observed using the JEOL JEM-6500 F field-emission scanning electron microscopy. The UV–Vis absorption spectra of the films were recorded using a JASCO V-650 spectrophotometer ranging from 300 to 900 nm. The composition of samples was determined using an energy dispersive spectroscopy (Horiba, EMAX-ENERGY) and a K-Alpha™ X-ray photoelectron spectrometer using an AlK α X-ray radiation source to estimate elements semi-quantitatively.

### Antibacterial experiment

Bacterial culturing and plating were performed following the previously described standard methods^5^,^47^,^48^,^49^. The bacterial concentration was determined using the standard plating method or from optical density readings at 595 nm (OD_595_). For example, the conversion factor for *E. coli* BL 21 was calculated to be 1 × 10^9^ colony-forming unit (CFU)/mL at OD_595_, and the cultures were diluted with the culture medium to 1 × 10^7^ CFU/mL. The 1 × 10^6^ CFU culture was allowed to drip on the sample (approximately 6.25 cm^2^) and was then placed in a dark room or exposed under the visible light and at room temperature. The visible light source was an incandescent lamp (Classictone incandescent lamp, 60 W, Philips Taiwan; Taipei, Taiwan), and the illumination density was recorded using a light meter (model LX-102; Lutron Electronic Enterprises, Taipei, Taiwan). In the photocatalytic reaction, the illumination distance between the sample and lamp was approximately 10 cm, which was exposed for 30 min, and the light intensity on the sample surface was nearly 1.2 × 103 lux (lumen/m^2^) (30 mW/cm^2^). After illumination, 100 μL of the bacterial solution was recovered from the sample. Finally, the bacterial concentration was determined using standard dilution and plating methods, and the percentage of surviving bacteria was calculated^18^,^19^. *S. pyogenes* (strain M29588), pandrug-resistant *A. baumannii* (strain M36788), and *S. aureus* (strain SA02) were the clinical isolates provided by Buddhist Tzu-Chi General Hospital in Hualien, Taiwan^5^. *E. coli* and *A. baumannii* were grown and maintained in the lysogeny broth (LB) medium or LB agar (BD Diagnostics, Sparks, MD, USA) at 37 °C. By contrast, *S. pyogenes* and *S. aureus* were grown in the trypticase soy broth with yeast extract (TSBY) medium or TSBY agar (MDBio, Inc., Taipei, Taiwan) at 37 °C. When the visible light was used to elicit the photocatalysis reaction, a UV cut-off filter (400 nm; Edmund Optics, Barrington, NJ, USA) was used to prevent the illumination of small fractions with UV-range wavelength in the photocatalytic experiments.

### Photocatalytic properties

Analysis methods were based on recently reported literatures^4^,^50^,^51^, which used the MB degradation rate to analyze the photocatalytic performance of impurity-doped TiO_2,_ in accordance with the generally accepted method and reported format of MB photodecomposition. Photocatalytic efficiency was evaluated by examining the decomposition of 10 ppm MB (Sigma-Aldrich, St. Louis, MO, USA). The MB concentration was determined according to the intensity of the light-absorption peak at 664 nm wavelength, as measured using a UV–Vis spectrometer. A fixed size (1 cm × 1 cm) of the sample sank in 2 mL of the MB aqueous solution. The visible light illumination was conducted using a fluorescent lamp (Philips; P-LF27W/865) with a wavelength distribution of approximately 400–750 nm and a maximum intensity at the range of 543–611 nm, producing an average power density of 4.2 mW/cm^2^ at a distance of 8 cm between the visible light source and sample.

### Statistical analysis

The means, standard deviations, and statistics of the quantifiable data were calculated using Microsoft Office Excel 2003, SigmaPlot 10, and SPSS 19. The significance of data was determined using a one-way analysis of variance followed by a post hoc Bonferroni corrected test. The probability of type I error α = 0.05 was recognized as statistically significant.

## Additional Information

**How to cite this article**: Wong, M.-S. *et al.* Antibacterial property of Ag nanoparticle-impregnated N-doped titania films under visible light. *Sci. Rep.* **5**, 11978; doi: 10.1038/srep11978 (2015).

## Supplementary Material

Supplementary Information

## References

[b1] McDonnell G. & Russell A. D. Antiseptics and disinfectants: activity, action, and resistance. Clinical microbiology reviews 12, 147–179 (1999). 9880479 10.1128/cmr.12.1.147 PMC88911

[b2] Liou J. W. & Chang H. H. Bactericidal effects and mechanisms of visible light-responsive titanium dioxide photocatalysts on pathogenic bacteria. Archivum immunologiae et therapiae experimentalis 60, 267–275 (2012). 22678625 10.1007/s00005-012-0178-x

[b3] Linsebigler A. L., Lu G. & Yates J. T. Photocatalysis on TiO_2_ surfaces: principles, mechanisms, and selected results. Chemical Reviews 95, 735–758 (1995).

[b4] Asahi R. *et al.* Visible-light photocatalysis in nitrogen-doped titanium oxides. Science 293, 269–271 (2001). 11452117 10.1126/science.1061051

[b5] Wong M. S. *et al.* Visible-light-induced bactericidal activity of a nitrogen-doped titanium photocatalyst against human pathogens. Applied and environmental microbiology 72, 6111–6116 (2006). 16957236 10.1128/AEM.02580-05 PMC1563686

[b6] Wong M. S., Sun D. S. & Chang H. H. Bactericidal performance of visible-light responsive titania photocatalyst with silver nanostructures. PLoS One 5, e10394 (2010). 20454454 10.1371/journal.pone.0010394 PMC2861596

[b7] Yu J. C. *et al.* Efficient visible-light-induced photocatalytic disinfection on sulfur-doped nanocrystalline titania. Environmental science & technology 39, 1175–1179 (2005). 15773492 10.1021/es035374h

[b8] Cheng C. L. *et al.* The effects of the bacterial interaction with visible-light responsive titania photocatalyst on the bactericidal performance. J Biomed Sci 16, 7 (2009). 19272171 10.1186/1423-0127-16-7 PMC2644973

[b9] Kau J. H. *et al.* Role of visible light-activated photocatalyst on the reduction of anthrax spore-induced mortality in mice. PLoS One 4, e4167 (2009). 19132100 10.1371/journal.pone.0004167 PMC2613519

[b10] Chang W. K. *et al.* Visible light responsive core-shell structured In_2_O_3_@CaIn_2_O_4_ photocatalyst with superior bactericidal property and biocompatibility. Nanomedicine : nanotechnology, biology, and medicine 8, 609–617 (2012). 10.1016/j.nano.2011.09.016 22033083

[b11] Chen Y. L. *et al.* The use of nanoscale visible light-responsive photocatalyst TiO_2_-Pt for the elimination of soil-borne pathogens. PLoS One 7, e31212 (2012). 22384003 10.1371/journal.pone.0031212 PMC3285157

[b12] Liou J. W. *et al.* Visible light responsive photocatalyst induces progressive and apical-terminus preferential damages on Escherichia coli surfaces. PLoS One 6, e19982 (2011). 21589873 10.1371/journal.pone.0019982 PMC3093399

[b13] Tseng Y. H. *et al.* Antibacterial performance of nanoscaled visible-light responsive platinum-containing titania photocatalyst *in vitro* and *in vivo*. Biochimica et biophysica acta 1830, 3787–3795 (2013). 23542693 10.1016/j.bbagen.2013.03.022

[b14] Fan J. C. C., Bachner F. J., Foley G. H. & Zavracky P. M. Transparent heat‐mirror films of TiO_2_/Ag/TiO_2_ for solar energy collection and radiation insulation Applied Physics Letters 25, 693–695 (1974).

[b15] Dhar A. & Alford T. L. High quality transparent TiO_2_/Ag/TiO_2_ composite electrode films deposited on flexible substrate at room temperature by sputtering. APL Materials 1, ID: 012102 (2013).

[b16] Goei R. & Lim T. T. Ag-decorated TiO_2_ photocatalytic membrane with hierarchical architecture: photocatalytic and anti-bacterial activities. Water research 59, 207–218 (2014). 24805373 10.1016/j.watres.2014.04.025

[b17] Zaknoon F. *et al.* Antibacterial properties of an oligo-acyl-lysyl hexamer targeting Gram-negative species. Antimicrobial agents and chemotherapy 56, 4827–4832 (2012). 22751534 10.1128/AAC.00511-12 PMC3421859

[b18] Wu M. S. *et al.* Nanodiamonds protect skin from ultraviolet B-induced damage in mice. Journal of nanobiotechnology 13, 35 (2015). 25947194 10.1186/s12951-015-0094-4 PMC4432518

[b19] Tauc J. Optical properties and electronic structure of amorphous Ge and Si. . Materials Research Bulletin 3, 37–46 (1968).

[b20] Chen Y. Y. *et al.* Surveillance on secular trends of incidence and mortality for device-associated infection in the intensive care unit setting at a tertiary medical center in Taiwan, 2000-2008: a retrospective observational study. BMC infectious diseases 12, 209 (2012). 22963041 10.1186/1471-2334-12-209 PMC3458996

[b21] Silver S., Phung le T. & Silver G. Silver as biocides in burn and wound dressings and bacterial resistance to silver compounds. J Ind Microbiol Biotechnol 33, 627–634 (2006). 16761169 10.1007/s10295-006-0139-7

[b22] Dunn K. & Edwards-Jones V. The role of Acticoat with nanocrystalline silver in the management of burns. Burns 30 Suppl 1, S1–9 (2004). 15327800 10.1016/s0305-4179(04)90000-9

[b23] Silver S. Bacterial silver resistance: molecular biology and uses and misuses of silver compounds. FEMS Microbiol Rev 27, 341–353 (2003). 12829274 10.1016/S0168-6445(03)00047-0

[b24] Sondi I. & Salopek-Sondi B. Silver nanoparticles as antimicrobial agent: a case study on E. coli as a model for Gram-negative bacteria. Journal of colloid and interface science 275, 177–182 (2004). 15158396 10.1016/j.jcis.2004.02.012

[b25] Morones-Ramirez J. R., Winkler J. A., Spina C. S. & Collins J. J. Silver enhances antibiotic activity against gram-negative bacteria. Science translational medicine 5, 190ra181 (2013). 10.1126/scitranslmed.3006276 PMC3771099 23785037

[b26] Choi J. Y. *et al.* Photocatalytic antibacterial effect of TiO(2) film formed on Ti and TiAg exposed to Lactobacillus acidophilus. J Biomed Mater Res B Appl Biomater 80, 353–359 (2007). 16850466 10.1002/jbm.b.30604

[b27] Hu C., Guo J., Qu J. & Hu X. Photocatalytic degradation of pathogenic bacteria with AgI/TiO_2_ under visible light irradiation. Langmuir : the ACS journal of surfaces and colloids 23, 4982–4987 (2007). 17373834 10.1021/la063626x

[b28] Hu C. *et al.* Ag/AgBr/TiO_2_ visible light photocatalyst for destruction of azodyes and bacteria. J Phys Chem B 110, 4066–4072 (2006). 16509698 10.1021/jp0564400

[b29] Matsui Y. *et al.* Effect of silver-carrying photocatalyst “Hikari-Gintech” on mycobacterial growth *in vitro*. Microbiol Immunol 48, 489–495 (2004). 15272193 10.1111/j.1348-0421.2004.tb03541.x

[b30] Yao Y. *et al.* Self-sterilization using silicone catheters coated with Ag and TiO_2_ nanocomposite thin film. J Biomed Mater Res B Appl Biomater 85, 453–460 (2008). 18098205 10.1002/jbm.b.30965

[b31] Caruso F. & Ariga K. Modern Techniques for Nano- and Microreactors/-reactions. Springer Science & Business Media (2010).

[b32] Mane S. T. *et al.* A study of nano crystalline Cd1-XCoXS thin composite films deposited by a liquid phase chemical bath deposition Advances in Applied Science Research 2, 8–18 (2011).

[b33] Kurtulus O., Daggumati P. & Seker E. Molecular release from patterned nanoporous gold thin films. Nanoscale 6, 7062–7071 (2014). 24842586 10.1039/c4nr01288g

[b34] Marambio-Jones C., Hoek EMV. A review of the antibacterial effects of silver nanomaterials and potential implications for human health and the environment. Journal of Nanoparticle Research 12, 1531–1551 (2010).

[b35] Batarseh K. I. & Smith M. A. Synergistic activities of a silver(I) glutamic acid complex and reactive oxygen species (ROS): a novel antimicrobial and chemotherapeutic agent. Current medicinal chemistry 19, 3635–3640 (2012). 22680634 10.2174/092986712801323216

[b36] Cai Y., Stromme M. & Welch K. Photocatalytic antibacterial effects are maintained on resin-based TiO_2_ nanocomposites after cessation of UV irradiation. PLoS One 8, e75929 (2013). 24146793 10.1371/journal.pone.0075929 PMC3798317

[b37] Carre G. *et al.* TiO_2_ photocatalysis damages lipids and proteins in Escherichia coli. Applied and environmental microbiology 80, 2573–2581 (2014). 24532071 10.1128/AEM.03995-13 PMC3993174

[b38] George S. *et al.* Differential effect of solar light in increasing the toxicity of silver and titanium dioxide nanoparticles to a fish cell line and zebrafish embryos. Environmental science & technology 48, 6374–6382 (2014). 24811346 10.1021/es405768n

[b39] Zou X., Shi J. & Zhang H. Coexistence of silver and titanium dioxide nanoparticles: enhancing or reducing environmental risks? Aquatic toxicology 154, 168–175 (2014). 24907921 10.1016/j.aquatox.2014.05.020

[b40] El-Badawy A., Feldhake D. & Venkatapathy R. State of the Science Literature Review: Everything Nanosilver and More. US Environmental Protection Agency. EPA/600/R–610/084 (2010).

[b41] Minetto D., Libralato G. & Volpi Ghirardini A. Ecotoxicity of engineered TiO_2_ nanoparticles to saltwater organisms: an overview. Environment international 66, 18–27 (2014). 24509165 10.1016/j.envint.2014.01.012

[b42] Klippstein R. *et al.* Silver nanoparticles interactions with the immune system: implications for health and disease. In: Nanotechnology and Nanomaterials (ed Perez D ). Intech (2010).

[b43] Juan L. *et al.* Deposition of silver nanoparticles on titanium surface for antibacterial effect. International journal of nanomedicine 5, 261–267 (2010). 20463942 10.2147/ijn.s8810 PMC2865021

[b44] Peng B. *et al.* Study on the Thermal Treatment of Nano-Ag/TiO_2_ Thin Film. ISRN Nanotechnology 2011, Article ID 614243 (2011).

[b45] Yu B. *et al.* Synthesis of Ag-TiO_2_ composite nano thin film for antimicrobial application. Nanotechnology 22, 115603 (2011). 21387845 10.1088/0957-4484/22/11/115603

[b46] Prasada R. G. S. V. *et al.* Nanostructured TiO_2_ and TiO_2_-Ag Antimicrobial Thin Films. In: Nanoscience, Technology and Societal Implications (NSTSI), 2011 International Conference. IEEE (2011).

[b47] Chang H. H. & Lo S. J. Modification with a phosphorylation tag of PKA in the TraT-based display vector of Escherichia coli. J Biotechnol 78, 115–122 (2000). 10725535 10.1016/s0168-1656(99)00227-8

[b48] Chang H. J., Sheu S. Y. & Lo S. J. Expression of foreign antigens on the surface of Escherichia coli by fusion to the outer membrane protein traT. J Biomed Sci 6, 64–70 (1999). 9933744 10.1007/BF02256425

[b49] Chang H. H., Shih K. N. & Lo S. J. Receptor-mediated endocytosis as a selection force to enrich bacteria expressing rhodostomin on their surface. J Biomed Sci 7, 42–50 (2000). 10644888 10.1007/BF02255917

[b50] Lin L. *et al.* Uniform carbon-covered titania and its photocatalytic property. Journal of Molecular Catalysis A: Chemical 236, 46–53 (2005).

[b51] Zhang H., Zong R., Zhao J. & Zhu Y. Dramatic visible photocatalytic degradation performances due to synergetic effect of TiO_2_ with PANI. Environmental science & technology 42, 3803–3807 (2008). 18546726 10.1021/es703037x

