# Supplementary material for: Antibacterial property of Ag nanoparticle-impregnated N-doped titania films under visible light
Source: Sci Rep. 2015 Jul 9;5:11978. doi: 10.1038/srep11978 (PMC4496671; doi:10.1038/srep11978)
Supplement: Supplementary Information [file srep11978-s1.doc]

**Supplemental Materials:**

**Antibacterial property of Ag nanoparticle-impregnated N-doped titania films under visible light**

**Ming-Show Wong**^1,2^, Chun-Wei Chen^1^, Chia-Chun Hsieh^1^, Shih-Che Hung^3^, Der-Shan Sun^3,4^ and Hsin-Hou Chang^2,3,4^*

Running title: Antibacterial multilayer TiO_2_-Ag/N films

Key words: TiO_2_, Ag and N co-doped TiO_2_, visible light responsive photocatalyst

**Page 1-5**: Supplementary Figure S1-S5.

**Page 6**: Supplementary Table 1.

**Page 7**: Supplementary Method.

**Figure S1**


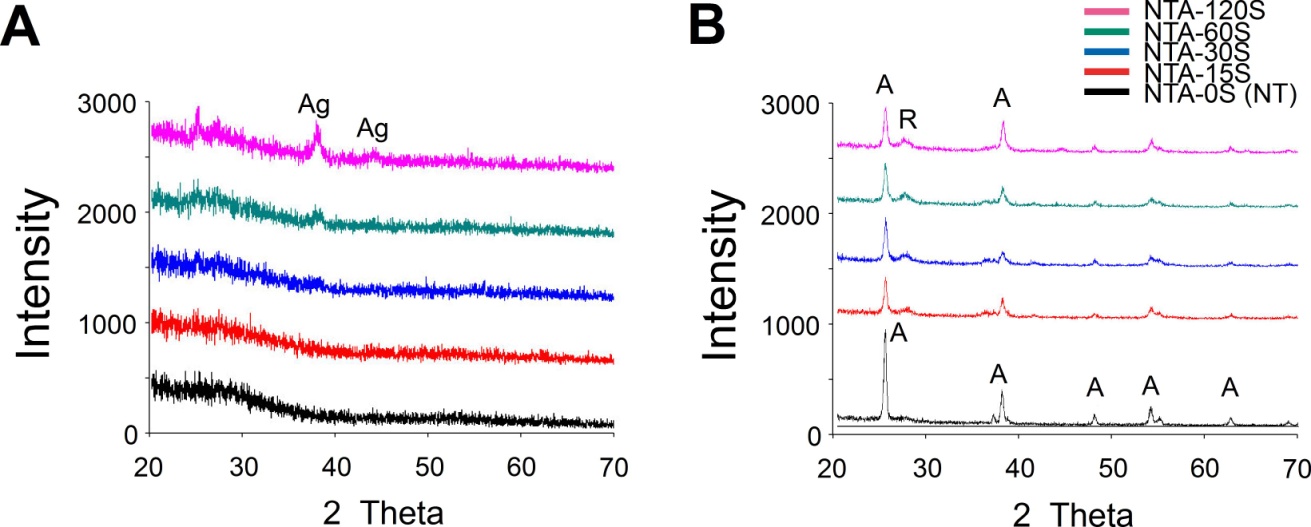


**Figure S1. Influence of silver content in TiO_2_(N)/Ag/TiO_2_(N) films.** The X-ray diffraction (XRD) analyses before (A) and after (B) annealing were shown. A: anatase; R: rutile (B). These results indicated that anatase of TiO_2_ was formed after the annealing.

**Figure S2**


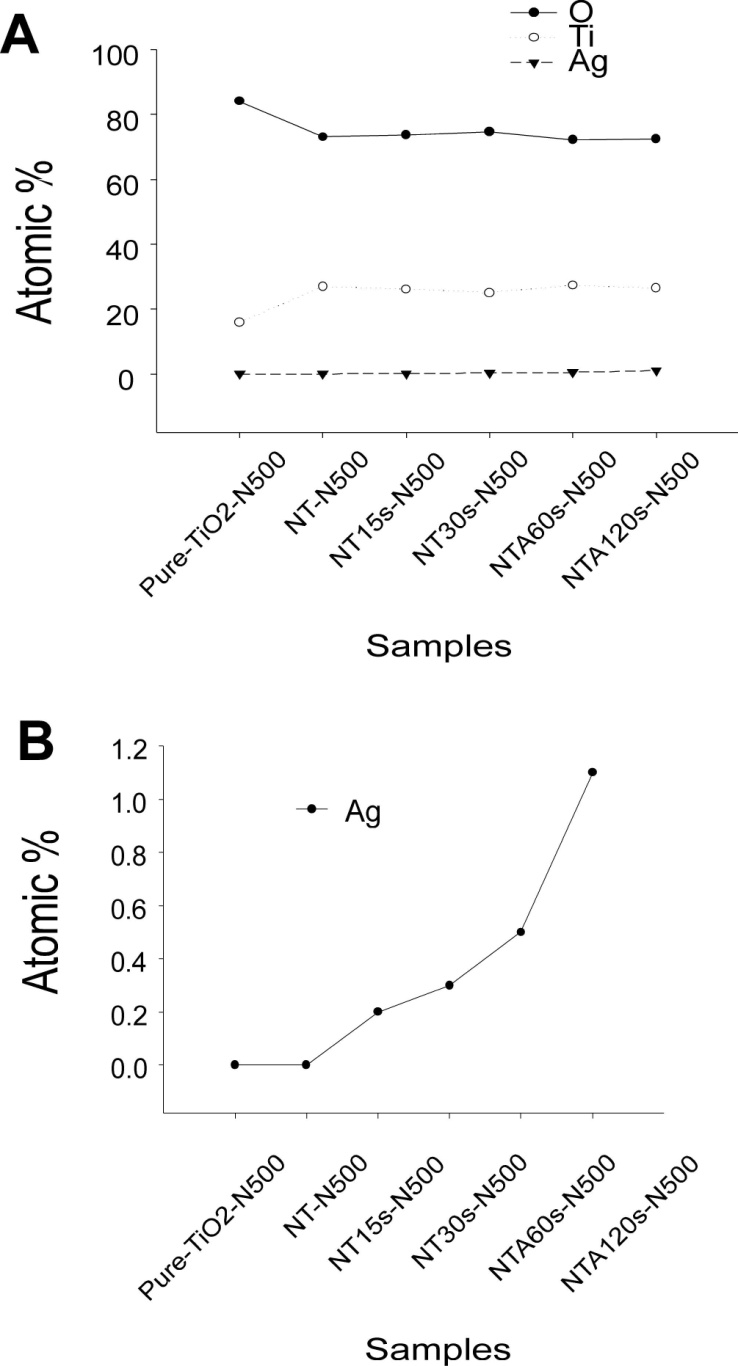


**Figure S2. The Energy-dispersive X-ray spectroscopy (EDS) analyses.** The EDS analyses of thin films with elements O, Ti Ag (A) or with Ag alone (B) indicated that deposition amount of Ag increased over time.

**Figure S3**


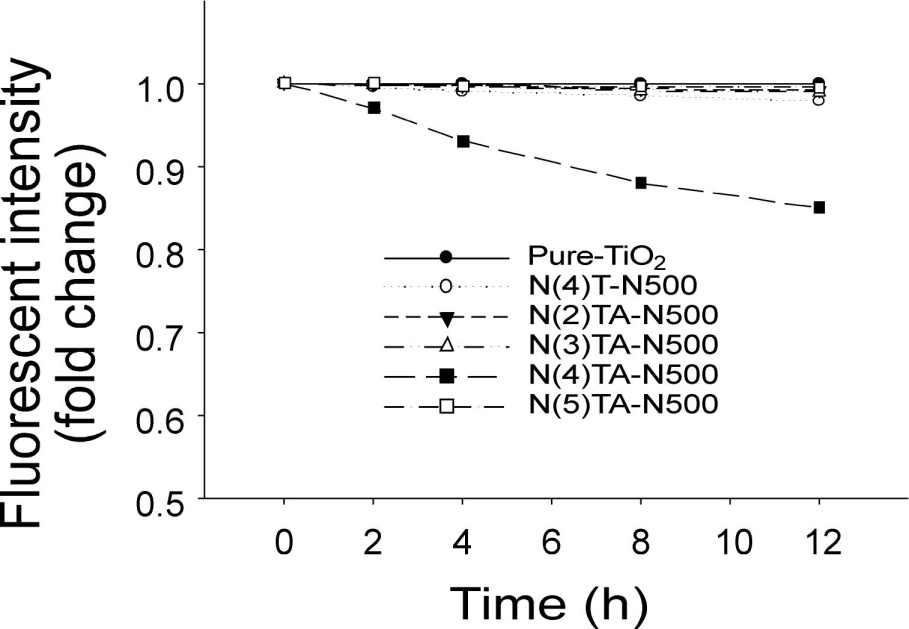


**Figure S3. Influence of nitrogen content in sandwich TiO_2_(N)/Ag/TiO_2_(N) films on the photocatalytic property.** The analysis results of photocatalysis-mediated degradation of fluorescent dye Hoechst 33258 were shown. The level of untreated groups (0 h) was normalized to 1 fold.

**Figure S4**


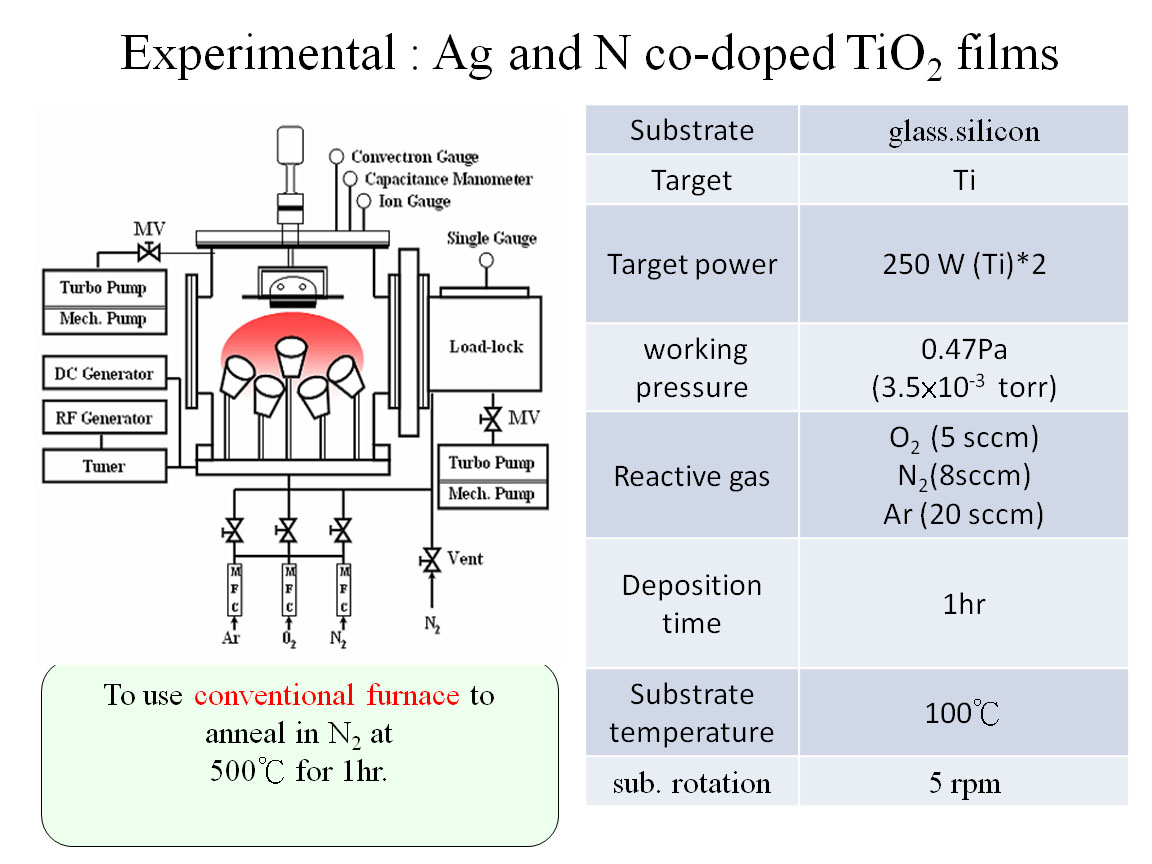


**Figure S4. Experimental settings.** The experimental settings of N doped TiO_2_ films.

**Figure S5**

**Figure S5. Experimental settings.** The schematic of Ag nanoparticle-impregnated N-doped titania films with different O_2_ supplies (2, 3, 4, 5 sccm).

**Table 1**

| **Sample ID** | **N(at%)** | **Band gap(eV)** |
| --- | --- | --- |
| Pure-TiO_2_-N500 | 0 | 3.4 |
| N(4)T-N500 | 4.36 | 3.1 |
| N(2)TA-N500 | 18.77 | – |
| N(3)TA-N500 | 13.47 | – |
| N(4)TA-N500 | 4.16 | 3.0 |
| N(5)TA-N500 | 3.47 | 3.0 |

**Supplementary Method:**

**Photocatalytic properties.** An alternative method using Hoechst 33258 (Sigma-Aldrich, St. Louis, MO, USA) as a target for analyzing photocatalysis performance of impurity doped TiO_2_ was also applied. The photocatalytic efficiency was measured by the decomposition of 0.1 µg/mL Hoechst 33258. The fixed size (1cm × 1cm) of photocatalyst samples sank in 2 ml of Hoechst 33258 aqueous solution. The visible-light illumination was carried out using a fluorescent lamp (Philips, P-LF27W/865) with the wavelength distribution around 400~750 nm and the maximum intensity at 543 and 611 nm, producing an average power density of 4.2 mW/cm^2^ at a distance of 8 cm between the Visible-light source and the sample. An UV cut-off filter (400 nm; Edmund Optics) was used to prevent illumination of small fractions of UV-range wave length during the visible-light elicited photocatalysis. The fluorescent intensity of Hoechst 33258, which correlated to the concentration, was finally examined using a fluorescent microplate reader through 350 nm excitation to result the light emission peak at 510 nm wavelength.
